# Tumor-associated macrophages promote cisplatin resistance in ovarian cancer cells by enhancing WTAP-mediated N6-methyladenosine RNA methylation via the CXCL16/CXCR6 axis

**DOI:** 10.1007/s00280-023-04533-8

**Published:** 2023-06-05

**Authors:** Lan Hong, Xiuzhen Wang, Lang Zheng, Shengtan Wang, Genhai Zhu

**Affiliations:** Department of Gynaecology, Hainan Affiliated Hospital of Hainan Medical University, No.19, Xiuhua Road, Xiuying District, Haikou, 570311 Hainan China

**Keywords:** Ovarian cancer, Tumor-associated macrophage, DDP resistance, CXCL16, N6-methyladenosine, WTAP

## Abstract

**Purpose:**

Tumor-promotive tumor-associated macrophages (TAMs) and the CXCL16/CXCR6 axis have been reported to be correlated with the limited efficacy of chemotherapy in ovarian cancer (OC). However, the role of TAM-secreted CXCL16 and the mechanism by which it affects the cisplatin (DDP) resistance of OC cells remain elusive.

**Methods:**

We induced human THP-1 monocytes to differentiate into macrophages. Next, SKOV3 and TOV-112D cells were co-cultured with the macrophages, followed by incubation with increasing concentrations of DDP. The effects of CXCL16, CXCR6, and WTAP on the DDP resistance of OC cells were investigated using the CCK-8 assay, colony formation assay, flow cytometry, and TUNEL staining. CXCL16 concentrations were determined by ELISA. Quantitative real-time PCR and western blotting were used to examine related markers.

**Results:**

Our results showed that after being co-cultured with TAMs, the DDP resistance of OC cells was significantly enhanced and their CXCL16 levels were elevated. Acquired DDP resistance was characterized by an increased IC_50_ value for DDP, the formation of cell colonies, and decreased levels of cell apoptosis, which were accompanied by reduced levels of caspase-3 and Bax expression, and increased levels of Bcl-2, PARP1, BRCA1, and BRCA2 expression. Either CXCL16 knockdown in TAMs or CXCR6 knockdown in OC cells suppressed the DDP resistance of OC cells that had been co-cultured with TAMs. Knockdown of CXCL16 affected m6A RNA methylation in OC cells, as reflected by decreased YTHDF1/WTAP expression and increased ALKBH5 expression. WTAP overexpression and knockdown promoted and suppressed the DDP resistance of OC cells, respectively.

**Conclusion:**

Tumor-associated macrophages promote the cisplatin resistance of OC cells by enhancing WTAP-mediated N6-methyladenosine RNA methylation via the CXCL16/CXCR6 axis.

**Supplementary Information:**

The online version contains supplementary material available at 10.1007/s00280-023-04533-8.

## Introduction

Ovarian cancer (OC) is the most frequently occurring gynecological malignancy and a major cause of death worldwide. The 5-year survival rate of OC patients is < 30% [1]. In the last three decades, platinum-based chemotherapy has become the first choice for treating the vast majority of advanced OC patients [2, 3]. Unfortunately, intrinsic and acquired resistance to platinum-based drugs, including cisplatin (DDP), remains a major clinical impediment [4]. As a consequence, it is important to explore DDP resistance-related molecular mechanisms and identify promising therapeutic targets for treating OC.

Recent studies have revealed the crucial roles played by the tumor microenvironment (TME) in tumor progression and drug resistance. One main component of the TME is tumor-associated macrophages (TAMs) [5, 6]. TAMs secrete cytokines into the TME; those cytokines include chemokine (C-X-C motif) ligand 16 (CXCL16), which comprises 254 amino acids and binds to CXC motif chemokine receptor 6 (CXCR6) [7, 8]. As opposed to other CXC chemokines, CXCL16, a member of the α-chemokine subfamily, is expressed as both a membrane-bound molecule and a soluble chemokine [9]. Previous studies have suggested that CXCL16 and CXCR6 are involved in the development and progression of various malignancies. For example, suppression of CXCL16 receptor CXCR6 was found to reduce tumor angiogenesis in a hepatocellular carcinoma xenograft mouse model [10]. Kim et al. [11] reported that targeting CXCL16 either in the microenvironment or cancer cells by use of shRNA efficiently blocked tumor growth and angiogenesis in thyroid cancers. Furthermore, CXCL16 promotes the development of gastric cancer by activating the ADAM10-dependent CXCL16/CXCR6 axis [12]. In a preliminary study, we found that soluble CXCL16 secreted by TAMs promoted SKOV3 cell migration and invasion by activating the NF-κB p65 and PI3K/AKT signaling pathways via upregulation of CXCR6 [13]. Interestingly, the CXCL16/CXCR6 axis participates in cancer chemoresistance, as shown by Kapur et al. [14], who demonstrated that the CXCR6–CXCL16 axis acts as a counter-defense mechanism and promotes docetaxel resistance in prostate cancer. Nevertheless, few studies have reported on the role played by macrophage-secreted CXCL16 in the DDP resistance of OC cells.

In recent years, N6-methyladenosine (m6A) RNA methylation has received increased scientific attention because of the key roles it plays in gene transcript expression, which is initiated by m6A methyltransferases as “writers,” recognized by m6A-binding proteins as “readers,” and removed by m6A demethylases as “erasers” in eukaryote cells [15]. The “writer” complex consists of methyltransferase-like 3 (METTL3), RNA-binding motif protein 15 (RBM15), Wilms tumor 1-associated protein (WTAP), zinc finger CCCH domain-containing protein 13 (ZC3H13), KIAA1429, and METTL14 [16]. The recognition process (“readers”) is mediated by m6A-binding proteins such as HNRNPA2B1, YTHDC1/2,YTHDF1/2/3, and LRPPRC [16]. Obesity-associated protein (FTO) [17] and alkB homolog 5 (ALKBH5) [18] make up the “eraser” complex, which stimulates the demethylation process. It has been reported that the expression levels of chemokines in cancer may be related to m6A levels in the cancer cells. Chen et al. [19] established a chemokine-related prognostic gene signature (CRPGS) model for prostate cancer, which was based on the expression levels of four chemokines (CXCL14, CCL20, CCL24, CCL26), and found that the CRPGS model was associated with m6A levels [19]. Another m6A signature-based risk prediction model for head and neck squamous cell carcinoma showed higher levels of chemokine and chemokine receptor expression in a high-risk group [20]. Moreover, modulation of m6A RNA modification is an important strategy for inhibiting chemoresistance during cancer treatment [21, 22]. For instance, eraser protein FTO was found to promote chemoresistance in cervical squamous cell carcinoma by demethylating β-catenin mRNA and stabilizing β-catenin [23]. Depletion of reader protein YTHDF1 was found to inhibit cell proliferation, xenograft tumor formation, and regulate the resistance of non-small cell lung cancer cells to DDP [24]. Therefore, it is worth exploring whether chemokines can modulate m6A levels and thereby regulate the cisplatin resistance in ovarian cancer.

In this study, we explored the effect of TAM-secreted CXCL16 on the DDP resistance of OC cells and further investigated whether CXCL16-mediated DDP resistance was correlated with changes in m6A methylation.

## Materials and methods

### Cell lines and treatment

Human monocyte cell line THP-1 and two OC cell lines (SKOV3 and TOV-112D) were provided by the ATCC (American Type Culture Collection, Manassas, VA, USA). To generate macrophages, THP-1 cells were cultured in 6-well plates (1 × 10^6^ per well) and then incubated with 320 nM phorbol 12-myristate 13-acetate (PMA, Sigma-Aldrich, Saint Louis, MO, USA) for 24 h [25]. OC cells were cultured in Dulbecco’s modified Eagle’s medium (Sigma‐Aldrich, USA) containing 10% fetal bovine serum (FBS) at 37 °C in a 5% CO_2_ atmosphere.

### Cell transfection

Three shRNAs targeting CXCL16 (shCXCL16), CXCR6 (shCXCR6), and WTAP (shWTAP), respectively, and their negative controls (shNC) were purchased from GeneChem Co., Ltd. (Shanghai, China). A plasmid that overexpressed human WTAP and a NC plasmid were provided by the Public Protein/Plasmid Library Company (Beijing, China). TAMs were transfected with shCXCL16 or the NC plasmid, while OC cells were transfected with shCXCR6, shWTAP or the WTAP overexpression plasmid for 48 h. All transfections were performed using Lipofectamine 3000 (Invitrogen, Carlsbad, CA, USA) according to the manufacturer’s instructions.

### Co-culture of TAMs and OC cells

Co-culture assays were conducted using Transwell chambers (0.4 µm pore size; Corning, Inc., NY, USA). Briefly, TAMs from the blank or transfection groups were seeded into the upper chamber and OC cells from the blank or transfection groups were plated into the lower chamber. After 24 h of incubation at 37 °C, the upper chamber was removed and OC cells in the lower chamber were harvested for use in subsequent experiments.

### DDP resistance assay

Cell Counting Kit‐8 (CCK-8) assays were performed to determine the DDP sensitivity of OC cells. In brief, SKOV3 and TOV-112D cells in the co-culture system were seeded into 96-well plates (3,000 cells per well) and treated with increasing concentrations of DDP (0, 0.5, 1, 2, 4, 8, and 16 μg/mL) for 24 h. After a 2 h incubation with 10 μL of CCK-8 reagent (Beyotime Institute of Biotechnology), the absorbance of each well at 450 nm was determined using a microplate reader, and the data were used to calculate the IC_50_ value of DDP.

### Colony formation assay

SKOV3 and TOV-112D cells were plated into six-well plates (800 cells per well) and treated with 2 μg/mL DDP for 24 h. After replacing the drug-containing medium with fresh medium, the cells were incubated for 1 week to obtain large cell colonies. Next, the colonies were fixed with methanol, stained with h 0.1% crystal violet for 30 min, and observed under a microscope.

### Apoptosis analysis by flow cytometry

SKOV3 and TOV-112D cells were treated with 2 μg/mL DDP for 24 h, harvested, and then washed three times with ice-cold PBS. Next, the cells were stained for 15 min with 5 µL of Annexin V-FITC dissolved in 1 × binding buffer, and then incubated for 5 min with 5 μL of propidium iodide (PI) dissolved in 120 μL of 1 × binding buffer under conditions of darkness. Finally, the percentages of apoptotic cells were determined using a FACScan flow cytometer (BD Biosciences, San Jose, CA, USA).

### Apoptosis analysis by TUNEL staining

TUNEL assays were performed to further assess apoptotic cells. Briefly, SKOV3 and TOV-112D cells were cultured in 12-well plates for 24 h, and then treated with 2 μg/mL DDP for 24 h. Next, the cells were collected, washed two times with PBS, and stained for 6 min with 50 µL of terminal Deoxynucleotidyl Transferase dUTP Nick End Labeling (TUNEL) solution at 37 °C in the dark. When observed under a fluorescence microscope, red fluorescence indicated an apoptotic cell and the cell nucleus exhibited blue fluorescence.

### Enzyme-linked immunosorbent assays (ELISA)

The SKOV3 and TOV-112D cellular supernatants of the co-culture system were harvested and then analyzed for their levels of CXCL16 using a Human CXCL16 ELISA kit (Westang Bio-Tech Co. Ltd, China) according to the manufacturer’s instructions.

### Quantification of m6A RNA

After extracting total RNA from SKOV3 and TOV-112D cells with TRIzol reagent (Invitrogen), the levels of m6A were examined using a EpiQuik™ m6A RNA methylation quantification kit (P-9005-48, Colorimetric, Epigentek, Farmingdale, NY, USA). Subsequently, the levels of m6A were quantified by reading the absorbance at 450 nm based on a standard curve.

### Quantitative real-time PCR

Total RNA was isolated using TRIzol reagent and cDNA was synthesized using a FastQuant RT Kit with gDNase (Tiangen, Beijing, China) according to instructions provided by the manufacturer. Next, quantitative real-time PCR was performed using a SYBR Green Kit (Thermo Fisher, Waltham, MA, USA) on an ABI PRISM 7300 Sequence Detection system (Applied Biosystems, Santa Clara, CA, USA). Relative gene expression was analyzed by the 2 − ΔΔCt method, and GAPDH served as a control. The nucleotide sequences of the primers are shown in Table S1.

### Western blot analysis

RIPA buffer (Beyotime) was used to extract the total cellular proteins and a BCA assay kit (Beyotime) was used to determine the protein concentration in each extract. A 40 μg sample of protein from each extract was separated by 10% SDS-PAGE and the protein bands were transferred onto polyvinylidene difluoride (PVDF) membranes (Millipore, Burlington, MA, USA), which were subsequently blocked with 5% non-fat milk in TBST for 2 h at room temperature. Next, the membranes were incubated with primary antibodies against caspase-3 (ab32042, Abcam, Cambridge MA, USA), Bax (ab182734, Abcam), Bcl-2 (ab182858, Abcam), PARP1 (ab227244, Abcam), BRCA1 (ab131360, Abcam), BRCA2 (ab239375, Abcam), YTHDF1 (ab220162, Abcam), WTAP (ab232392, Abcam), ALKBH5 (ABE547, Sigma-Aldrich), and β-actin (ab8227, Abcam) at 4 °C overnight, followed by a 1.5 h incubation with a horseradish peroxidase (HRP)-conjugated secondary antibody at room temperature. Finally, the target proteins were visualized with an enhanced chemiluminescence kit (Pierce, Waltham, MA, USA).

### Statistical analysis

All data were analyzed using GraphPad Prism 8.0 software (GraphPad Software, San Diego, CA, USA), and results are expressed as a mean value ± standard deviation (SD). Differences between two independent groups were analyzed by the Student’s *t* test and differences among three groups were analyzed by one-way analysis of variance (ANOVA) followed by Tukey’s test. A *p* value < 0.05 was considered to be statistically significant.

## Results

### Co-culture with TAMs induced DDP resistance in OC cells

**Fig. 1:**
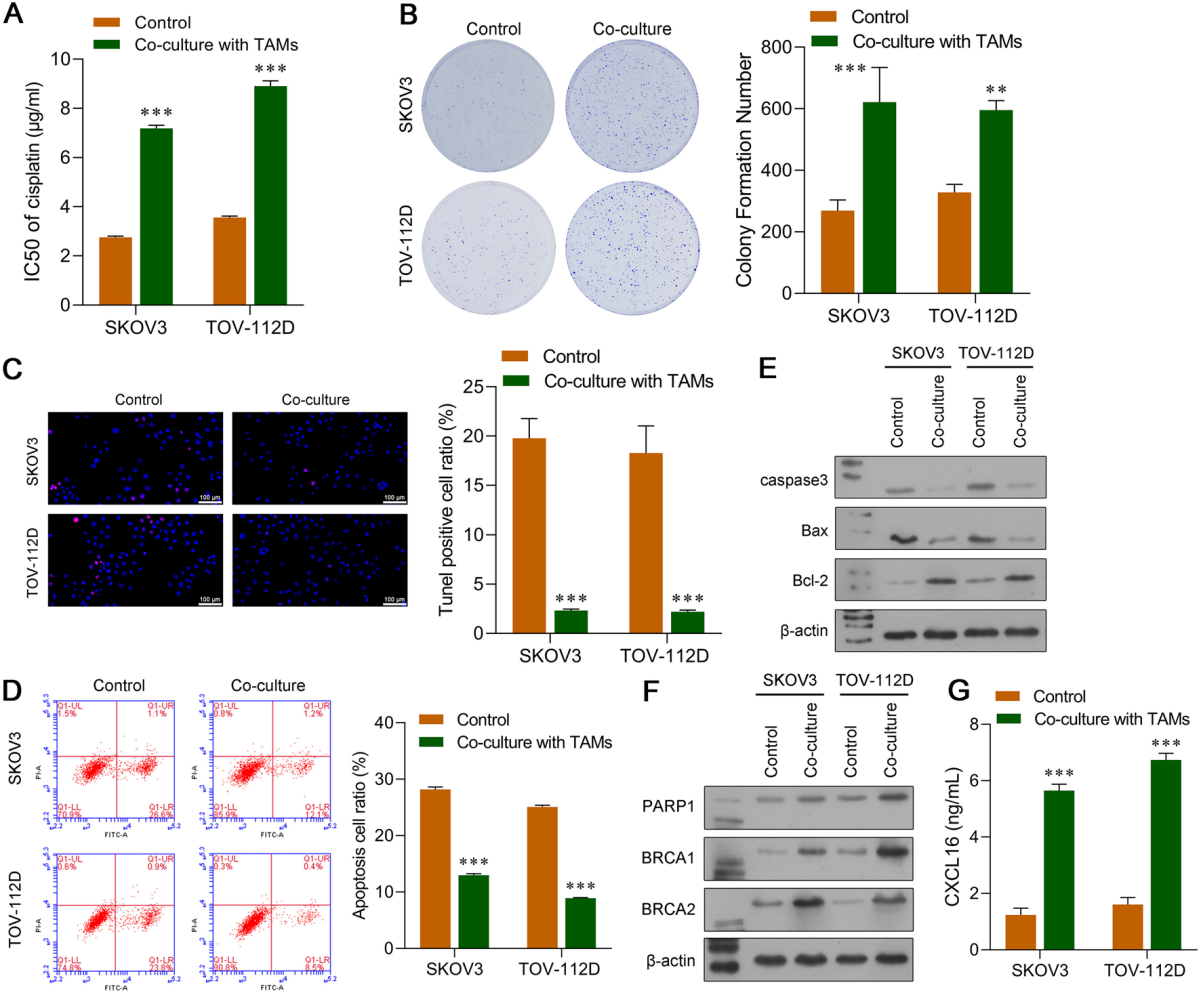
Co-culture with TAMs induced DDP resistance in OC cells. **A** CCK-8 assays were conducted to determine the IC_50_ values of DDP in SKOV3 and TOV-112D cells that were co-cultured with TAMs. **B** The colony formation assay, **C** TUNEL staining, and **D** flow cytometry were used to analyze the viability and apoptosis of SKOV3 and TOV-112D cells that were co-cultured with TAMs treated with 2 μg/mL DDP. Proteins associated with apoptosis **E** and DNA repair **F** were examined by western blotting. **G** The concentrations of CXCL16 in SKOV3 and TOV-112D cells co-cultured with TAMs were determined by ELISA. Data represent the mean value ± SD of three separate assays. ***p < 0.001, compared with control

To evaluate whether TAMs were involved in the DDP resistance of OC, we co-cultured SKOV3 and TOV-112D cells with TAMs in a non-contact Transwell system. Next, CCK-8 assays for cell viability were performed to determine the IC_50_ values of DDP. As indicated in Fig. 1A, the IC_50_ values of DDP significantly increased when DDP was used to treat SKOV3 and TOV-112D cells that had been co-cultured with TAMs (*p* < 0.001). Moreover, after being treated with 2 μg/mL DDP, the colony numbers of TAM co-cultured SKOV3 and TOV-112D cells were obviously increased (Fig. 1B). Both TUNEL staining (Fig. 1C) and flow cytometry (Fig. 1D) revealed that SKOV3 and TOV-112D cells co-cultured with TAMs had significantly lower rates of apoptosis than OC cells that were cultured alone. We next examined the levels of proteins associated with apoptosis and DNA repair. As shown in Fig. 1E, the levels of pro-apoptotic proteins (caspase-3 and Bax) were downregulated, and the levels of anti-apoptotic protein Bcl-2 were upregulated in SKOV3 and TOV-112D cells that had been co-cultured with M2-like TAMs. As shown in Fig. 1F, the levels of DNA repair-related proteins (PARP1, BRCA1, and BRCA2) were all obviously increased in SKOV3 and TOV-112D cells that had been co-cultured with TAMs. In addition, ELISA results showed that the levels of CXCL16 were notably upregulated in SKOV3 and TOV-112D cells co-cultured with TAMs when compared to the levels in OC cells cultured alone.

### Knockdown of CXCL16 in TAMs suppressed DDP resistance in co-cultured OC cells

**Fig. 2:**
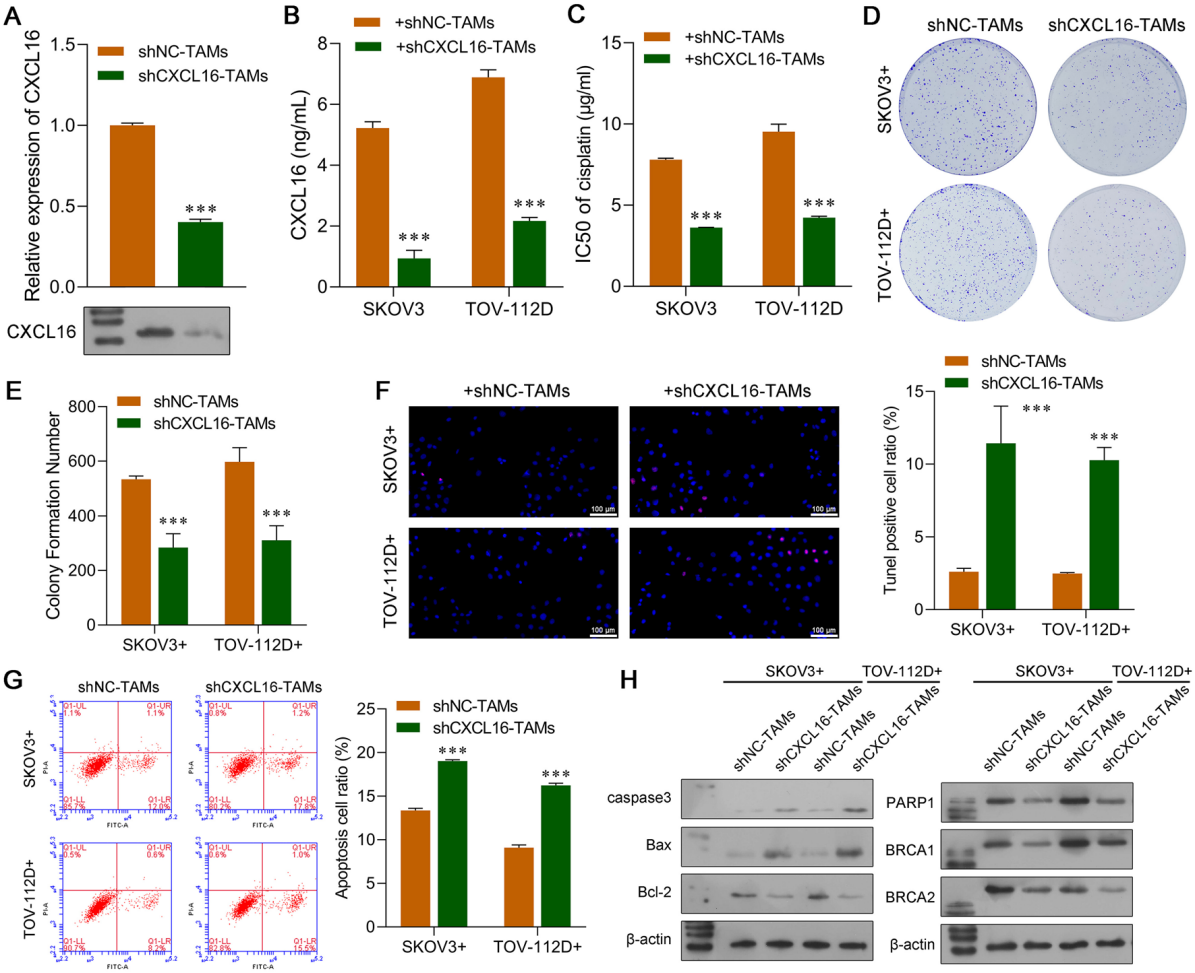
Knockdown of CXCL16 in TAMs suppressed DDP resistance in co-cultured OC cells. TAMs were transfected with shCXCL16 or shNC and then co-cultured with SKOV3 or TOV-112D cells. **A** CXCL16 expression in TAMs was determined using quantitative real-time PCR and western blot analysis. **B** ELISA assays were conducted to analyze CXCL16 concentrations in the co-culture system. **C** CCK-8 assays were performed to determine the IC_50_ values of DDP in SKOV3 and TOV-112D cells co-cultured with CXCL16-silenced TAMs that were treated with different concentrations of DDP. **D** and **E** The colony formation assay, **F** TUNEL staining, and **G** flow cytometry were used to analyze the viability and apoptosis of SKOV3 and TOV-112D cells were co-cultured with CXCL16-silenced TAMs treated with 2 μg/mL DDP. **H** Proteins associated with apoptosis and DNA repair were examined by western blotting. Data represent the mean value ± SD of 3 separate assays. ***p < 0.001, compared with shNC-TAMs

To confirm the role of CXCL16 secreted by TAMs, loss-of-function assays were conducted with TAMs, which were then co-cultured with OC cells. Quantitative real-time PCR and western blot studies confirmed the inhibition of CXCL16 in TAMs (Fig. 2A). After CXCL16 knockdown, the concentrations of CXCL16 were significantly reduced in the co-culture environment of TAMs and OC cells (Fig. 2B). After treatment with increasing concentrations of DDP, the IC_50_ value of DDP was obviously decreased in the SKOV3 and TOV-112D cells that had been co-cultured with CXCL16-silenced TAMs (Fig. 2C). Moreover, knockdown of CXCL16 in TAMs significantly decreased colony formation (Fig. 2D and E) and promoted cell apoptosis (Fig. 2F and G) in SKOV3 and TOV-112D cells treated with 2 μg/mL DDP. At the protein level, the levels of apoptotic-related caspase-3 and Bax were upregulated, while Bcl-2 was downregulated in SKOV3 and TOV-112D cells co-cultured with CXCL16-silenced TAMs, when compared with control TAMs (Fig. 2H). Additionally, three different.

DNA repair-related proteins (PARP1, BRCA1, and BRCA2) were downregulated after CXCL16 knockdown in SKOV3 and TOV-112D cells co-cultured with TAMs (Fig. 2H).

### Knockdown of CXCR6 in OC cells decreased DDP resistance in the co-culture system

**Fig. 3:**
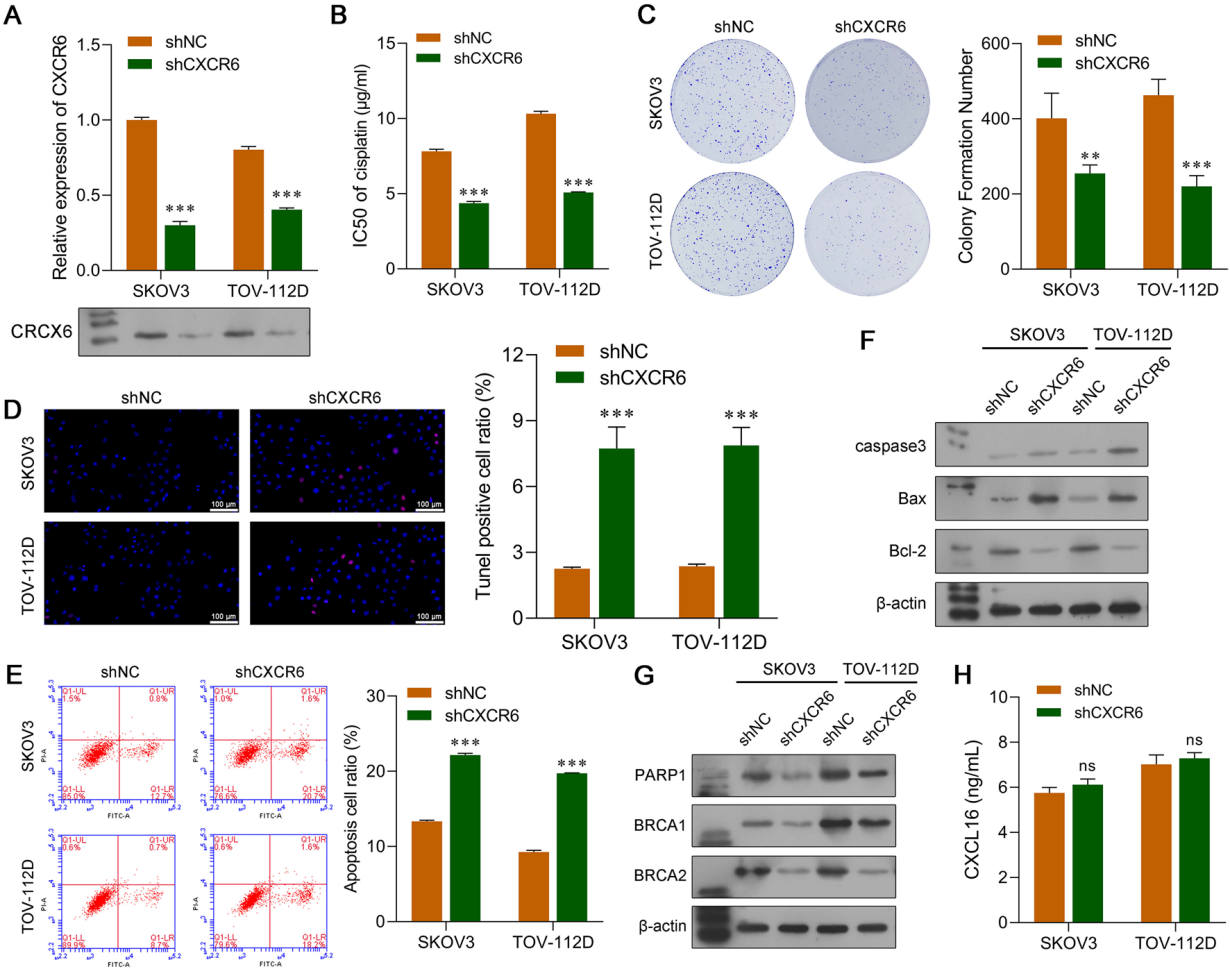
Knockdown of CXCR6 in OC cells decreased DDP resistance in the co-culture system. SKOV3 and TOV-112D cells were transfected with shCXCR6 or the shNC, followed by co-culture with TAMs. **A** Quantitative real-time PCR and western blotting were used to confirm the downregulation of CXCR6 expression in SKOV3 and TOV-112D cells transfected with shCXCR6. **B** CCK-8 assays were performed to determine the IC_50_ values of DDP in CXCR6-silenced SKOV3 and TOV-112D cells co-cultured with TAMs that were treated with different concentrations of DDP. **C** The colony formation assay, **D** TUNEL staining, and **E** flow cytometry were used to analyze the viability and apoptosis of CXCR6-silenced SKOV3 and TOV-112D cells co-cultured with TAMs that were treated with 2 μg/mL DDP. Proteins associated with apoptosis **F** and DNA repair **G** were examined by western botting. **H** ELISA assays were conducted to analyze the concentration of CXCL16 in the co-culture system. Data represent the mean value ± SD of three separate assays. ***p < 0.001, compared with shNC

After confirming the role of chemokine CXCL16 in TAMs, we next explored the functional role of CXCR6 as a CXCL16 receptor in OC cells in the co-culture system. Our data showed that the levels of CXCR6 mRNA and protein in SKOV3 and TOV-112D cells were significantly reduced after those cells were transfected with shCXCR6 (Fig. 3A). Moreover, knockdown of CXCR6 markedly decreased the IC_50_ values of DDP in SKOV3 and TOV-112D cells co-cultured with TAMs (Fig. 3B). After treatment with 2 μg/mL DDP, CXCR6 silencing notably suppressed colony formation (Fig. 3C) and promoted cell apoptosis (Fig. 3D and E) in SKOV3 and TOV-112D cells co-cultured with TAMs. Western blot analyses showed that SKOV3 and TOV-112D cells obtained from the co-culture system had increased levels of caspase-3 and Bax expression and lower levels of Bcl-2 expression after CXCR6 knockdown (Fig. 3F). Transfection with shCXCR6 suppressed PARP1, BRCA1, and BRCA2 protein expression in SKOV3 and TOV-112D cells from the co-culture system (Fig. 3G). ELISA results showed that CXCL16 concentrations in the shNC and shCXCR6 groups of SKOV3 and TOV-112D cells from the co-culture system were not significantly different (Fig. 3H).

### Knockdown of CXCL16 in TAMs affected m^6^A RNA methylation in OC cells

**Fig. 4:**
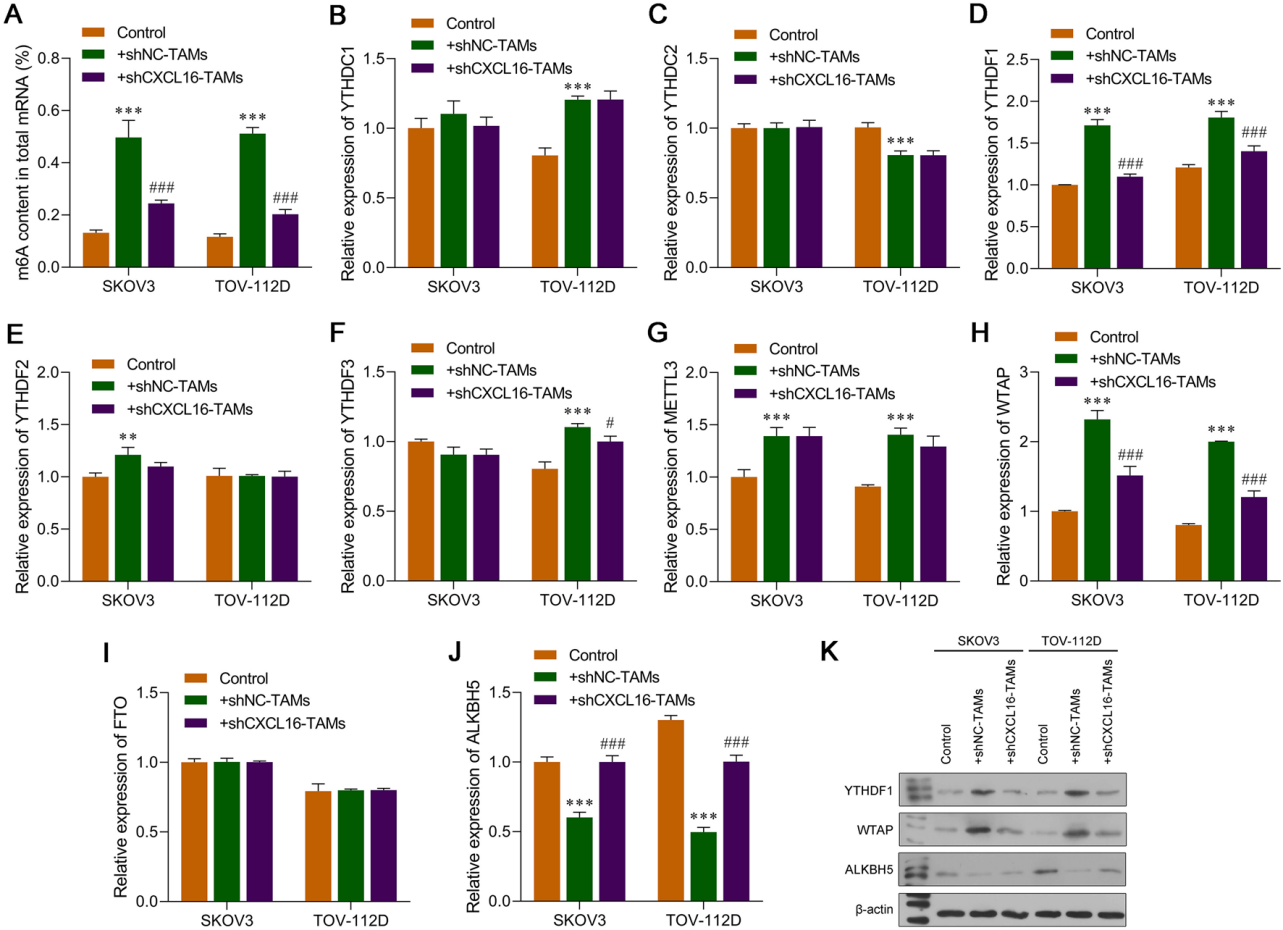
Knockdown of CXCL16 in TAMs affected m^6^A RNA methylation in OC cells. TAMs were transfected with shCXCL16 or shNC and then co-cultured with SKOV3 and TOV-112D cells. **A** The amount of m6A in the total RNA of SKOV3 and TOV-112D cells was measured using an EpiQuik m6A RNA methylation quantification kit. **B**–**J** The expression levels of mRNA for m6A-related methyltransferases (METTL3 and WTAP), demethylases (FTO and ALKBH5), and binding proteins (YTHDC1/2 and YTHDF1/2/3) in SKOV3 and TOV-112D cells were determined by quantitative real-time PCR. **K** Western blot analyses were performed to verify significant changes in the expression of m6A-related proteins (YTHDF1, WTAP, and ALKBH5) in SKOV3 and TOV-112D cells. Data represent the mean value ± SD of three separate assays. **p < 0.01, ***p < 0.001, compared with control; #*p* < 0.05, ###*p* < 0.001, compared with shNC-TAMs

Because m6A participates in tumor-related drug resistance, we investigated whether CXCL16 helped regulate m6A methylation in OC cells. We first measured the levels of m6A in the total RNA of SKOV3 and TOV-112D cells. As shown in Fig. 4A, the levels of m6A in the total RNA of SKOV3 and TOV-112D cells were significantly increased by co-culture with TAMs and decreased by CXCL16 silencing in TAMs. Subsequently, we determined the levels of m6A-related methyltransferases, demethylases, and binding proteins. Based on results from quantitative real-time PCR studies (Fig. 4B–J), we verified that the levels of m6A-related binding protein YTHDF1 and m6A-related methyltransferase WTAP were upregulated, while those of m6A-related demethylase ALKBH5 were downregulated in both SKOV3 and TOV-112D cells after co-culture with TAMs when compared to those levels in OC cells that were cultured alone, which were reversed after OC cells were co-cultured with CXCL16-silenced TAMs. The results from western blot studies were consistent with those from qRT-PCR assays (Fig. 4K). Notably, m6A-related methyltransferase WTAP showed the greatest change in expression and was selected for subsequent experiments.

### WTAP-mediated m^6^A RNA methylation promoted the DDP resistance of OC cells

**Fig. 5:**
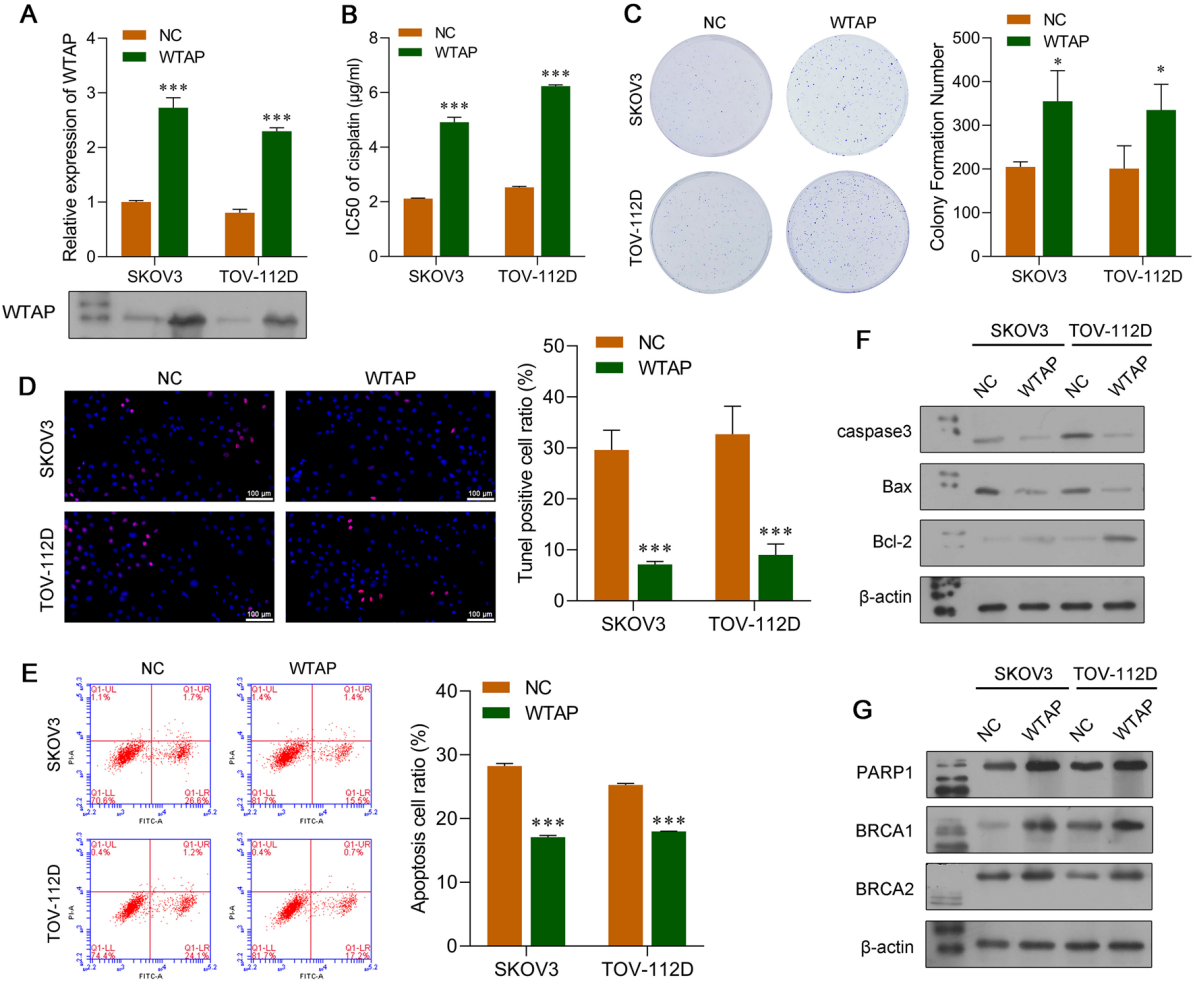
Overexpression of WTAP promoted the DDP resistance of OC cells. SKOV3 and TOV-112D cells were transfected with a WTAP overexpression plasmid or NC. **A** Quantitative real-time PCR and western blot analyses were performed to confirm the overexpression of WTAP in SKOV3 and TOV-112D cells. **B** CCK-8 assays were performed to determine the IC_50_ value of DDP in WTAP-overexpressing SKOV3 and TOV-112D cells that had been co-cultured with TAMs treated with different concentrations of DDP. **C** The colony formation assay, **D** TUNEL staining, and **E** flow cytometry were used to analyze the viability and apoptosis of WTAP-overexpressing SKOV3 and TOV-112D cells that were co-cultured with TAMs. Proteins associated with apoptosis **F** and DNA repair **G** were examined by western blotting. Data represent the mean value ± SD of three separate assays. ***p < 0.001, compared with NC

**Fig. 6:**
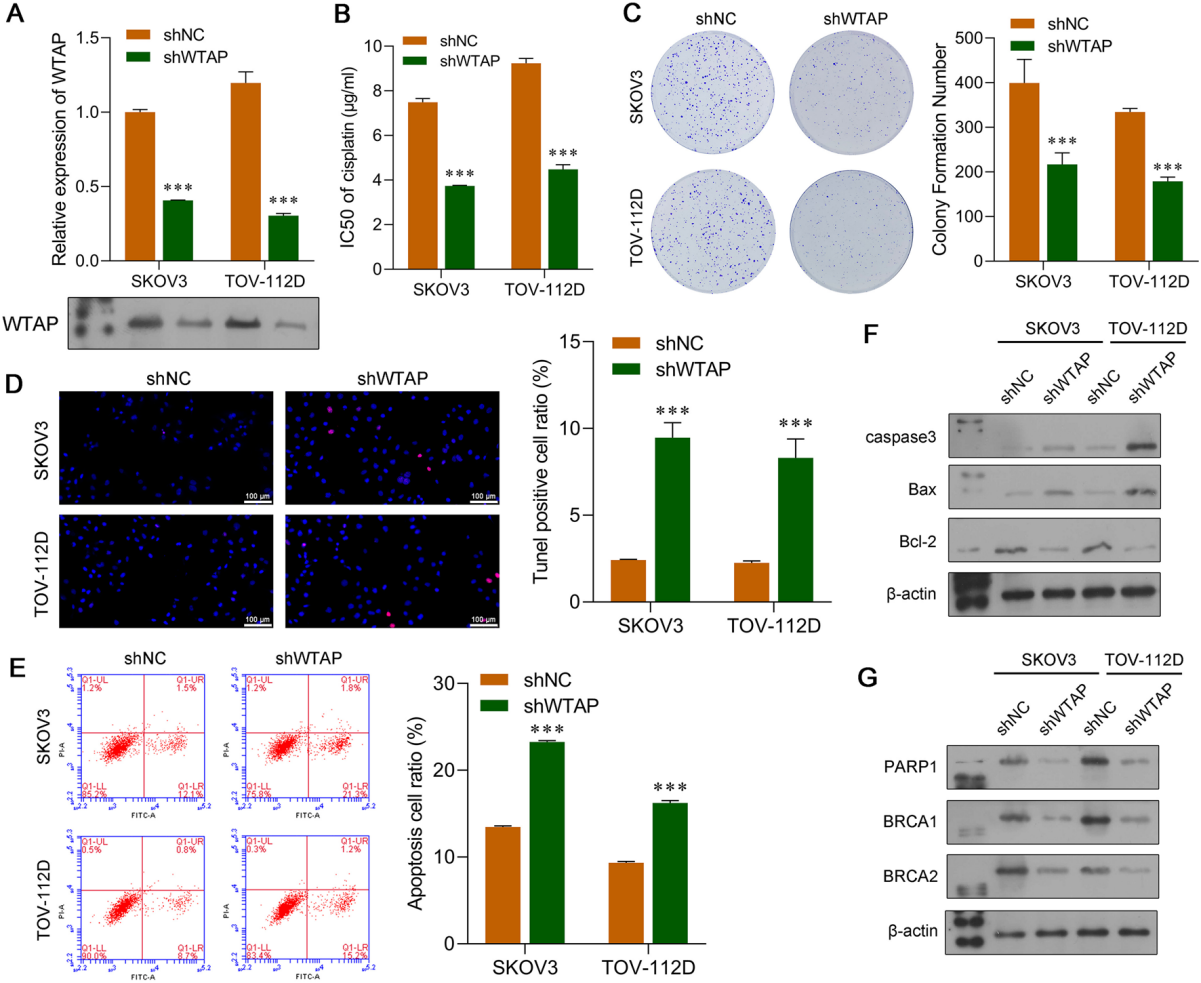
Knockdown of WTAP suppressed the DDP resistance of OC cells. SKOV3 or TOV-112D cells were transfected with shWTAP or shNC, followed by co-culture with TAMs. **A** Quantitative real-time PCR and western blot analyses were performed to confirm the downregulation of WTAP in SKOV3 and TOV-112D cells. **B** CCK-8 assays were performed to determine the IC_50_ value of DDP in WTAP-silenced SKOV3 and TOV-112D cells co-cultured with TAMs that were treated with different concentrations of DDP. **C** The colony formation assay, **D** TUNEL staining, and **E** flow cytometry were used to analyze the viability and apoptosis of WTAP-silenced SKOV3 and TOV-112D cells co-cultured with TAMs. Proteins associated with apoptosis **F** and DNA repair **G** were examined by western blotting. Data represent the mean value ± SD of three separate assays. ***p < 0.001, compared with shNC

Because CXCL16 knockdown decreased the expression of m6A-related methyltransferase WTAP, we speculated that WTAP might play a key role in regulating the DDP resistance of OC cells co-cultured with TAMs. To test this hypothesis, we performed gain-of-function and loss-of-function assays with SKOV3 and TOV-112D cells. WTAP overexpression plasmids were transfected into OC cells. After confirming the overexpression of WTAP in SKOV3 and TOV-112D cells (Fig. 5A), a chemosensitivity assay showed increased IC_50_ values in the SKOV3 and TOV-112D cells with WTAP overexpression (Fig. 5B). Moreover, WTAP overexpression increased colony formation (Fig. 5C) and decreased the cell apoptosis (Fig. 5D and E) induced by DDP in SKOV3 and TOV-112D cells. Western blot analyses showed that overexpression of WTAP suppressed DDP-induced apoptosis (reduced caspase-3 and Bax levels; increased Bcl-2 levels) (Fig. 5F) and enhanced the DNA repair capability (increased PARP1, BRCA1, and BRCA2 levels) of SKOV3 and TOV-112 cells (Fig. 5G). Loss-of-function assays showed that WTAP expression in OC cells was inhibited by shRNA; after which, the OC cells were co-cultured with TAMs and treated with DDP. In those studies, knockdown of WTAP was confirmed at both the mRNA and protein levels (Fig. 6A). A subsequent series of functional experiments showed that knockdown of WTAP decreased the IC_50_ value of DDP (Fig. 6B), decreased cell colony formation (Fig. 6C), and increased the rate of apoptosis (Fig. 6D and E). Furthermore, the expression levels of protein markers associated with apoptosis were consistent with results from TUNEL and flow cytometric analyses (Fig. 6F), while DNA repair-related proteins (PARP1, BRCA1, and BRCA2) were all inhibited by shWTAP (Fig. 6G).

## Discussion

TAMs are considered to be critical for establishing an intricate cellular communication network between cancer cells and their microenvironments over the course of tumorigenesis [26]. TAMs generally exhibit a pro-tumorigenic phenotype in the OC microenvironment, and are characterized by high levels of cytokine and chemokine expression [27]. Hence, forcing an interruption of pro-tumor signaling within the OC resistance–TAM communication network is a promising strategy for treating OC. It is a rational approach to find novel targets involved in pro-tumor signaling based on the sustainability of the tumor microenvironment. Accumulating evidence suggests that CXCL16 could be such a target because it affects tumor cell proliferation. It has been shown that overexpression of CXCL16 enhances the proliferation of TPC-1 and K-1 papillary thyroid cancer cells [28]. Furthermore, the CXCR6–CXCL16 axis promotes cell proliferation and docetaxel resistance in prostate cancer [14]. These reports support our observation that DDP resistance was elevated in OC cells that had been co-cultured with TAMs, and that effect was accompanied by upregulated CXCL16 levels.

This OC–TAMs communication system can be interrupted by elimination of CXCL16 expression by use of a gene silencing technique. Here, we showed that transfection of TAMs with shCXCL16 significantly reduced the DDP resistance of OC cells. In fact, the role of CXCL16 in tumorigenesis is closely associated with tumor-correlated cells, which suggests that CXCL16 acts on tumor-associated cells and is thus secreted into the tumor microenvironment [29]. Moreover, knockdown of CXCR6 as the CXCL16 receptor in OC cells also decreased DDP resistance in our co-culture system. Consistent with those findings, our previous study showed that TAM-secreted CXCL16 contributes to OC cell migration and invasion by upregulating CXCR6 expression [13]. Similarly, other cytokine genes have also been found to mediate communication between TAMs and cancer cells. For example, TAMs are recruited to glioma cells and produce CXCL7, which promotes the stemness of glioma cells [30]. In breast cancer, TAM-secreted CCL2 promotes an endocrine resistance by activating the PI3K/AKT/mTOR signaling pathway [31]. These findings indicate the importance of TAM-mediated DDP resistance in regulating the CXCL16/CXCR6 axis.

To further explore the mechanism by which CXCL16 promotes OC cell acquired resistance to DDP, we focused on RNA m6A modification, which is one of the important regulators of cell function. We found a higher m6A level in OC cells that had been co-cultured with TAMs, and that effect was reversed by CXCL16 inhibition in TAMs. Additional studies indicated that the increased levels of m6A in OC cells induced by co-culture with TAMS were accompanied by increased levels of YTHDF1/WTAP expression and decreased levels of ALKBH5 expression. Moreover, a suppression of m6A by CXCL16 inhibition was accompanied by decreased YTHDF1/WTAP expression and slightly increased ALKBH5 expression. Although only limited knowledge exists concerning the association between CXCL16/CXCR6 and m6A modification, some studies on how m6A modification regulates drug resistance in the TME have been reported. For example, Lan et al. [32] reported that TAMs promoted METTL3-mediated m6A modification to enhance oxaliplatin resistance in colorectal cancer cells. In hepatocellular carcinoma, depletion of METTL3 under hypoxic conditions promoted sorafenib resistance and increased the expression levels of angiogenesis-related genes in cultured hepatoma cells [33]. Knockdown of the m6A demethylase gene (*ALKBH5*) enhanced the sensitivity of tumor cells to immunotherapy [34]. Herein, we found that WTAP overexpression and knockdown promoted and suppressed the DDP resistance of OC cells, respectively. Likewise, a disruption of WTAP expression was found to decrease the resistance of nasal-type natural killer/T cell lymphoma (NKTCL) cells to chemotherapy with DDP, as demonstrated by decreased expression of drug resistance-associated protein [35]. Finally, WTAP promoted multiple chemotherapy resistance and radiotherapy resistance in gastric cancer [36].

**Fig. 7:**
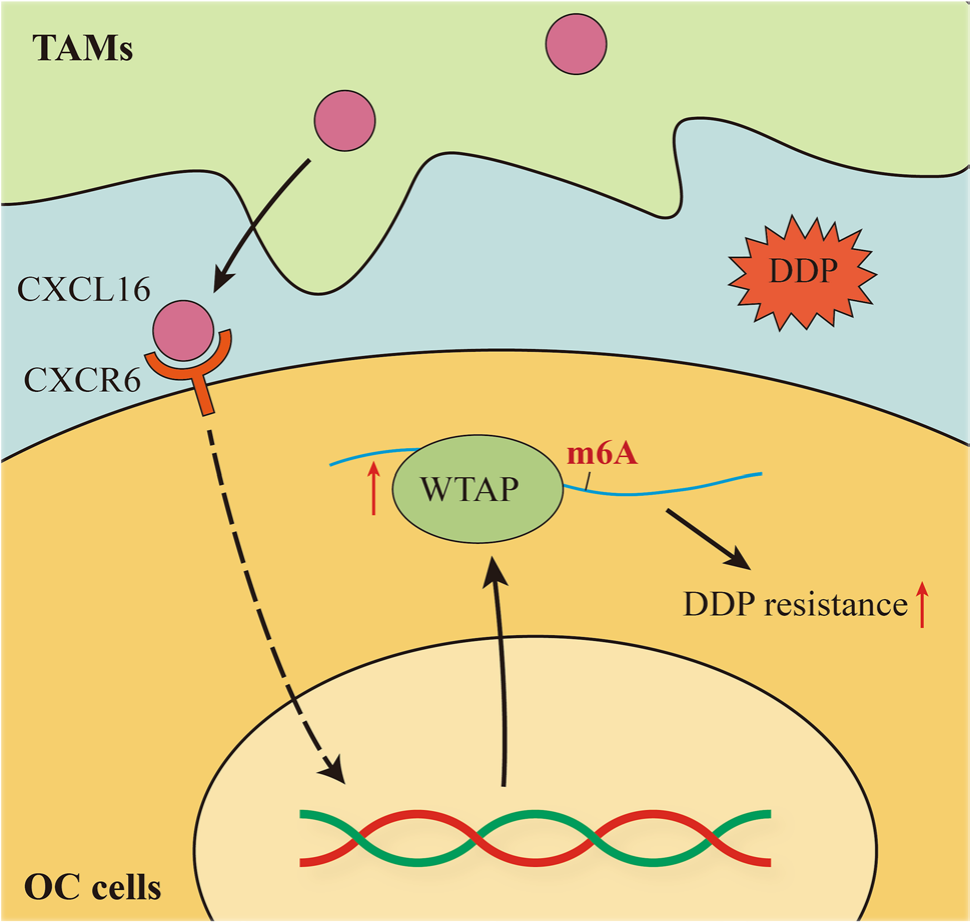
TAMs promoted DDP resistance in OC cells by enhancing WTAP-mediated N6-methyladenosine RNA methylation via the CXCL16/CXCR6 axis

In summary, our findings revealed a novel cellular crosstalk mechanism between TAMs and OC cells that was mediated by the CXCL16/CXCR6 axis. This crosstalk can regulate WTAP-mediated m6A RNA methylation to promote the DDP resistance of OC cells (Fig. 7). Targeting of CXCL16/WTAP-mediated m6A RNA methylation may be a novel strategy for enhancing the treatment response of DDP-resistant OC.

## Supplementary Information

Below is the link to the electronic supplementary material.

Supplementary file1 (DOCX 16 KB)

## Data Availability

All data generated or analyzed in this research are available in the published article.
