# Supplementary material for: Tumor-associated macrophages promote cisplatin resistance in ovarian cancer cells by enhancing WTAP-mediated N6-methyladenosine RNA methylation via the CXCL16/CXCR6 axis
Source: Cancer Chemother Pharmacol. 2023 Jun 5;92(1):71–81. doi: 10.1007/s00280-023-04533-8 (PMC10261262; doi:10.1007/s00280-023-04533-8)
Supplement: Supplementary file 1 — Supplementary file1 (DOCX 16 KB) [file 280_2023_4533_MOESM1_ESM.docx]

**Table 1. Primer pairs used for quantitative RT-PCR analysis.**

| **Gene ID** | **Sequence (5’- 3’)** |
| --- | --- |
| GAPDH F | TGTTCGTCATGGGTGTGAAC |
| GAPDH R | ATGGCATGGACTGTGGTCAT |
| CXCL16 F | AGTAGGATCACTGTCCTCGG |
| CXCL16 R | TGCACAGCACATAGGAAAGG |
| YTHDF1 F | TGGACACCCAGAGAACAAAAG |
| YTHDF1 R | TGTCCAGTAAGGTAGGGCTC |
| YTHDF2 F | GCAGTGGGTTCGGTCATAAT |
| YTHDF2 R | GGAACGATAAGCAGCATCCA |
| YTHDF3 F | CTTACCGTTCCCTGAATGGG |
| YTHDF3 R | TGCCCTTCCACTTATCCTGA |
| YTHDC1 F | CAGGAAGTGGACAGACGATT |
| YTHDC1 R | GGTGTGGAGGTTGTTCCATT |
| YTHDC2 F | CCAAGACGATTGGCAGCTAT |
| YTHDC2 R | TGTCTTTGGAGAAACCCTGC |
| METTL3 F | CCAGGGTCTGGATTGTGATG |
| METTL3 R | TGGGTACCTTTGCTTGAACC |
| METTL14 F | TTGTCTTGGTAGAAGACGCC |
| METTL14 R | TAGATTTGGGAGGAGGCGAT |
| WTAP F | GACGTCTGGGTCTGGATTTC |
| WTAP R | CCTTTCCCACTCACTGCTTT |
| FTO F | CCCGAACATTACCTGCTGAT |
| FTO R | TTCCAGAAGCTGACCTCTGA |
| ALKBH5 F | GTTCAAGCCTATTCGGGTGT |
| ALKBH5 R | GCTCAGGGACTTTGTTTCCA |
| CXCR6 F | TGTTTGTCTGTGGTCTGGTG |
| CXCR6 R | GGATGAGCATGGACGTGTAG |

F: forward primer; R: reverse primer.
